# The importance of sialic acid, pH and ion concentration on the interaction of uromodulin and complement factor H

**DOI:** 10.1111/jcmm.16492

**Published:** 2021-03-31

**Authors:** Lufeng Bai, Qiuyu Xie, Min Xia, Kunjing Gong, Na Wang, Yuqing Chen, Minghui Zhao

**Affiliations:** ^1^ Renal Division Department of Medicine Peking University First Hospital Beijing China; ^2^ Peking University Institute of Nephrology Beijing China; ^3^ Key Laboratory of Renal Disease Ministry of Health of China Beijing China; ^4^ Key Laboratory of Chronic Kidney Disease Prevention and Treatment Ministry of Education Beijing China; ^5^ Peking‐Tsinghua Center for Life Sciences Beijing China

**Keywords:** complement factor H, pH, sialic acid, sodium, Uromodulin

## Abstract

Uromodulin (UMOD) can bind complement factor H (cFH) and inhibit the activation of complement alternative pathway (AP) by enhancing the cofactor activity of cFH on degeneration of C3b. UMOD, an N‐glycans‐rich glycoprotein, is expressed in thick ascending limb of Henle's loop where the epithelia need to adapt to gradient change of pH and ion concentration. ELISA‐based cofactor activity of cFH and erythrocytes haemolytic assay was used to measure the impact of native and de‐glycosylated UMOD on the functions of cFH. The binding assay was performed under different pH and ion concentrations, using ELISA. The levels of sialic acid on UMOD, from healthy controls and patients with chronic kidney disease (CKD), were also detected by lectin‐ELISA. It was shown that removal of glycans decreased the binding between UMOD and cFH and abolished the ability of enhancing C3b degradation. In acidic condition, the binding became stronger, but it reduced as sodium concentration increased. A significant decrease of α‐2,3 sialic acids on UMOD was observed in CKD patients compared with that of healthy individuals. The sialic acids on UMOD, local pH and sodium concentration could impact the binding capacity between UMOD and cFH and thus regulate the activation of complement AP.

## 1 INTRODUCTION

Uromodulin (UMOD, also called Tamm‐Horsfall protein) has been gradually recognized as a protective factor and a biomarker of kidney injury. Although UMOD has been found for over 60 years, its new functions are constantly being discovered. UMOD has been recognized as an important regulatory protein of innate immune via binding complement fragments.^1^, ^2^, ^3^, ^4^, ^5^, ^6^ Our previous study has shown that UMOD can bind complement factor H (cFH) and enhance its ability as a cofactor of complement factor I to degenerate C3b.^7^

UMOD is expressed and secreted by epithelial cell on thick ascending limb (TAL) of Henle's loop and early distal convoluted tubules (DCT) of the nephron,^8^ which are important functional segments of renal tubules.^7^, ^9^, ^10^ There are gradient changes of pH and ion concentration in this part under physiological and pathological status,^11^, ^12^ and the epithelial cells in the region are sensitive to the changes of pH and ion concentration as well as the state of hypoxia and ischaemia.^13^, ^14^, ^15^ UMOD is also a highly glycosylated protein, about 28% of its molecular weight is carbohydrates. There are seven actual N‐glycosylation sites, among which Asn275 carries one high‐mannose chain, and the other six sites are composed of di‐, tri‐ and tetra‐antennary types of N‐linked glycans.^16^ Sialic acids are the outermost properties of the glycan chains. cFH has specific binding sites to sialic acids,^17^, ^18^ and sialic acid–bound cFH has 10‐fold affinity for C3b compared with C3b to its activator.^19^, ^20^

Thus, we explored the impact of pH, ion concentration and sialic acids on the interaction of UMOD and cFH.

## 2 MATERIALS AND METHODS

### 2.1 CKD cohort

In this study, we recruited 17 healthy volunteers and 36 patients with chronic kidney disease (CKD). The average age of the 36 CKD patients was 38.0 ± 12.6 years. The renal diseases included IgA nephropathy, lupus nephritis, ischemic renal disease, ANCA‐associated vasculitis, diabetic nephropathy, Alport syndrome, thrombotic microangiopathy, hypertensive kidney disease and glomerulonephritis. The average serum creatinine was 212.0 ± 102. 6 μmol/L (Table 1). We collected their urine, purified the UMOD to exclude the influence of proteinuria and same dosage of UMOD were used in the experiments to avoid effect of UMOD level from different patients.

**TABLE 1:** Clinical characteristics of the control and CKD groups

	Control group	CKD group	*P* value
Numbers	17	36	−
Age (year)	31.7 ± 9.9	38.0 ± 12.6	.10
Gender (male/female)	7/10	17/19	.68
Creatinine serum (μmol/L)	NA	212.0 ± 102.6	−
eGFR (mL/min per 1.73 m^2^)	NA	36.49 ± 32.05	−

Note

Data are normally distributed and presented as the mean ± SEM. The t‐test was used to compare between groups. CKD diagnosis was based on KDIGO guidelines. The 36 CKD patients included 19 IgA nephropathy, 5 lupus nephritis, 2 ischemic renal disease, 2 ANCA‐associated vasculitis, 2 diabetic nephropathy, 2 Alport syndrome, 1 thrombotic microangiopathy, 1 hypertensive kidney disease and 2 chronic glomerulonephritis.

Abbreviations: ANCA: anti‐neutrophil cytoplasmic antibody; CKD: chronic kidney disease; eGFR: estimated glomerular filtration rate.

### 2.2 Uromodulin purification

UMOD was purified according to protocol from previous study.^21^ Briefly, 1‐L urine was mixed with 20 g diatomaceous earth (Celite 521, Acros Organics) for 20 minutes at 4℃ and the mixture was transferred into a funnel with filter paper (Whatman, 15 cm diameter). After filtration, the layer of diatomaceous earth was washed with 0.025 mol/L sodium‐phosphate buffer and then mixed with deionized water for 30 minutes at 4℃, and centrifuged at 20 000 g for 30 minutes at 4℃. The 0.1 mol/L phosphate buffer and NaCl were added into the supernatant to the final concentration of 0.0025 and 0.14 mol/L, respectively. After mixing for 5 minutes, 5‐g diatomaceous earth was added, mixed for 20 minutes at 4℃. The mixture was transferred into a funnel with filter paper (Whatman, 7 cm diameter). After filtration, the layer of diatomaceous earth was washed with PBS and taken out to mix with deionized water for 30 minutes at 4℃ and centrifuged at 20 000 ×g for 30 minutes at 4℃. The supernatant was collected for dialysis in deionized water at 4℃ for overnight. After dialysis, the supernatant was concentrated by centrifugation at 3000 rpm using 30 000 NMWL filter (Amicon^TM^ Ultra‐15; Milipore), and then UMOD was lyophilized and kept at −80℃ before the experiment.

The purity of UMOD was evaluated with silver stain and western blot (Figure S1). Purified UMOD was separated in 10% SDS‐PAGE gel. After electrophoresis, the gel was silver stained following the procedure described in silver staining kit (Coolaber, Beijing). Purified UMOD was boiled with 5 x loading buffer for 10 minutes and separated in 10% polyacrylamide gel by SDS‐PAGE. Proteins were then transferred to polyvinylidene difluoride (PVDF) membranes and blocked for 1 hours in 5% non‐fat milk. Then, purified UMOD was detected by polyclonal antibody to human cFH (Cloud‐Clone Corp.) and polyclonal antibody to human complement factor I (Cloud‐Clone Corp.). After washing, the secondary horseradish peroxidase antibody was used. The membranes were exposed and analysed in GE Image Quant LAS 4000 chemiluminescence imaging analyser.

### 2.3 Cofactor activity of cFH

The measurement of cofactor activity of cFH followed previous study.^7^ Briefly, the experiment was performed in fluid mixture 20 μL, which included cFH (0.5 μg, Merck, Kenilworth), factor I (50 ng, Merck), C3b (3 μg, Merck) and UMOD (8 μg). The mixture was incubated at 37℃ for 10, 20, 30 and 40 minutes, respectively. C3b and its cleavage products were detected by western blotting with a polyclonal anti‐C3c antibody (Dako Cytomation).

### 2.4 Haemolytic assay

The assay was modified according to previous study.^22^ Sera were from three healthy individuals. Positive control was 100% haemolysis of sheep erythrocytes mixed with 6 μL of mouse anti‐human cFH monoclonal antibody (BioPorto Diagnostics A/S) and the normal serum. 30 μL of serum was mixed with cFH antibody, incubated at 4℃ for 1 hours, and then 5, 10 and 20 μg of UMOD were added to the mixtures, respectively. 3 μg of commercial cFH was added. At last, the mixtures were diluted in alternative pathway (AP) buffer (consist of 144 mmol/L NaCl, 7 mmol/L MgCl_2_, 2.5 mmol/L barbital, 1.5 mmol/L sodium barbital and 10 mmol/L EGTA) to total volume of 100 μL. The samples of each group were prepared in duplicate. The blank of each group was prepared by diluted samples in AP buffer plus 50 mmol/L EDTA. Final assay was carried out by adding 100 μL of sheep erythrocytes (5 × 10^6^ cells/mL in AP buffer) to the mixtures and incubated at 37℃ for 30 minutes. Then the samples were centrifuged at 5000 rpm for 5 minutes. The supernatants were transferred and the absorbance at 414 nm (A414 nm) was determined. The haemolysis (%) was calculated by subtraction of A414 nm of the samples and their corresponding blank dividing the A414 nm of total lysis (100 μL sheep erythrocytes and 100 μL deionized water).

### 2.5 Removal of glycan chains

The PNGase F (P0704) and α2‐3,6,8,9 Neuraminidase A (P0722) were purchased from New England BioLabs Inc The experiments were carried out according to the manufacturer's instructions with some modifications. N‐glycan was removed both under denatured condition and native condition. Under denatured condition, UMOD was mixed with glycoprotein denaturing buffer, boiled for 10 minutes at 100℃, and cooled down on ice. And then, Glycobuffer 2, NP‐40, and PNGase F were added to the mixture and incubated at 37℃ for overnight. Under native condition, UMOD, GlycoBuffer 2 and PNGase F were directly mixed and incubated at 37℃ for overnight. Sialic acids were removed by α2‐3,6,8,9 Neuraminidase A in a mixture of UMOD and GlycoBuffer 1, and incubated at 37℃ overnight. After incubation, the mixtures were subjected to western blotting to verify the removal of carbohydrates. And then, the mixtures were transferred to 30,000 NMWL filter (Amicon^TM^ Ultra‐15, Milipore) and the buffers were replaced with water by centrifugation at 3000 rpm at 4℃ for 10 times. Then the protein concentration was determined by NanoDrop 1000.

### 2.6 Binding of uromodulin with cFH by ELISA

These experiments were performed according to the previous study.^7^ Shortly, 96‐well plates were coated with cFH (4 μg/mL, in 100 mmol/L carbonate buffer, 100 μL per well, pH 9.6) at 4℃ for overnight. Because of interference of THP due to its viscidity of plastic plates, we coated 96‐wells plates with cFH. The results have been proved to be convincing by our previous study. After incubation, plates were washed three times with 10 mmol/L PBS with 0.1% Tween‐20 (PBST). The reaction volume was 100 μL and incubation was at 37℃ for 1 hours. Different concentrations of UMOD (controls, filtered and not filtered PNGase F‐treated, neuraminidase‐treated and THP with neuraminidase) were added. After washing, bound‐UMOD was detected by rabbit anti‐human UMOD polyclonal antibody (Biomedical Technologies Inc). Then alkaline phosphatase‐conjugated secondary anti‐rabbit IgG antibody (Sigma‐Aldrich) was used. Finally, absorbance was read at OD405nm.

### 2.7 Binding of complement factor H and uromodulin under different pH and concentration of sodium

This assay was performed according to previous study.^7^ The 96‐cells plate was first coated with 4 μg/mL cFH in 100 mmol/L carbonate buffer (100 μL per well, pH 9.6) at 4℃ for overnight. Then the 96‐cells plate was blocked by 1% bovine serum albumin (BSA) at 37℃ for 1 hour. Different concentrations (0, 2, 4, 8 μg/mL) of THP were added. The mixture was incubated in binding buffer with different pH (4 to 9) and various concentrations of sodium (50, 100, 150, 200 mmol/L) at 37°C for 1 hour respectively. After incubation, the plate was washed with PBST (300 μL/cell) for three times. Then the plate was incubated with PBST diluted rabbit anti‐human UMOD polyclonal antibody (Santa Cruz Biotechnology, USA) at 37℃ for 1 hour and then goat anti‐rabbit IgG alkaline phosphatase (Sigma Aldrich) was used. After washed, the 96‐cells plate was filled with alkaline phosphatase substrate (Amresco) for 20 minutes. The OD at 405 nm was measured by a microplate reader (iMark, BIO‐RAD). The binding buffer was based on PBS, pH was adjusted by HCl or NaOH, and sodium concentration was adjusted by changing the dosage of NaCl in PBS buffer. An artificial urine was also used to repeat the above experiments. The artificial urine (200 mmol/L Urea, 1.0 mmol/L Uric acid, 4.0 mmol/L Creatinine, 5.0 mmol/L Na_3_C_3_H_5_O_7_, 54.0 mmol/L NaCl, 30.0 mmol/L KCl, 3.0 mmol/L CaCl_2_, 2.0 mmol/L MgSO_4_, 2.0 mmol/L NaHCO_3_, 0.1 mmol/L NaC_2_O_4_, 9.0 mmol/L Na_2_SO_4_, 0.4 mmol/L Na_2_HPO_4_, 3.6 mmol/L NaH_2_PO_4_) was prepared according to previous report.^23^

### 2.8 Measurement of sia‐α (2,3) Gal/GalNAc with Lectin‐ELISA

Sia‐α (2,3) Gal/GalNAc was specifically recognized by biotinylated Maackia ameurensis lectin II (MAL II from Vector Laboratories). 96‐well plates were coated with UMOD (1 μg/mL, in 50 mmol/L carbonate buffer, 100 μL per well, pH 9.6) at 4℃ for overnight. After being washed with 0.1% PBST for four times, the plates were filled with 1x Carbo‐Free^TM^ Blocking Solution (Vector Laboratories) for 1 hour at 37℃. After washing, MAL II diluted into 10 μg/mL (in 1% BSA) was added to the plates and incubated at 37℃ for 1 hour. Then the plates were washed four times and filled with horseradish peroxidase (HRP)‐conjugated streptavidin (Thermo Fisher Scientific) for 1 hour at 37℃. Then the plates were washed and filled with chemiluminescent substrate (Thermo Fisher Scientific) for 10 min, and the reaction was stopped by 1 mol/L H_2_SO_4_. Finally, absorbance was read at OD450/570 nm.

### 2.9 Statistical analyses

Statistical software SPSS 14.0 (SPSS) was used for statistical analysis. Quantitative data were expressed as mean ± SEM. For normally distributed data, one‐way ANOVA was used for comparison of continuous data. For data which did not assume normal distribution, nonparametric tests were used to compare data significance. Statistical significance was considered as *P* < .05.

## 3 RESULTS

### 3.1 Uromodulin strengthened the function of complement factor H

**FIGURE 1:**
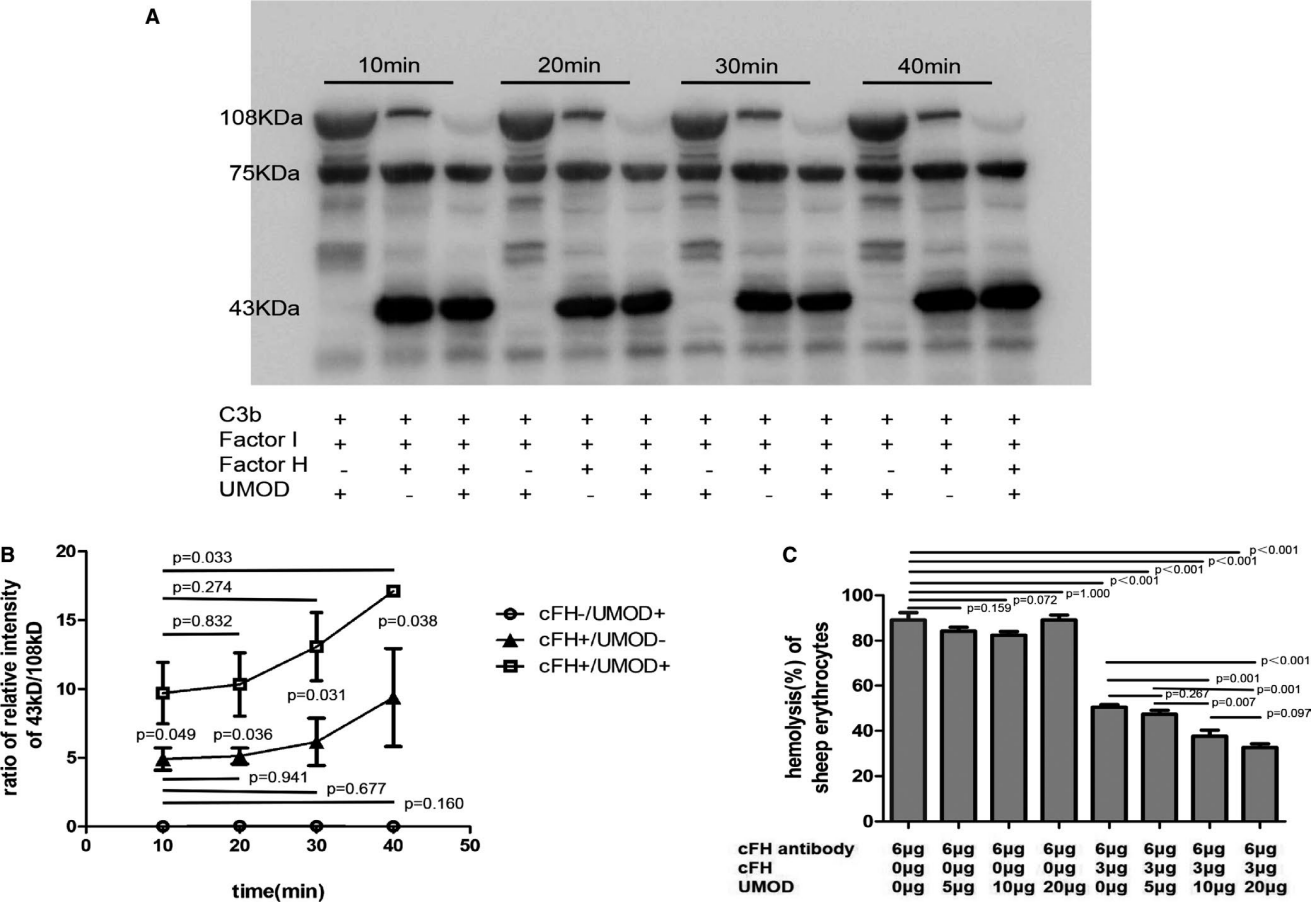
UMOD‐cFH binding enhanced the function of cFH. A, Uromodulin (UMOD)‐cFH binding accelerated the degradation of C3b by Western blotting. The cofactor activity of factor H was assayed in the fluid phase. The fluid reaction system including C3b (3 μg), cFI (50 ng) and cFH (0.5 μg), without or with UMOD (8 μg) were incubated and samples were collected at 10, 20, 30 and 40 min, respectively. The control group included C3b (3 μg), cFI (50 ng) and UMOD (8 μg) were also collected at 10, 20, 30 and 40 min, respectively. The experiments were repeated at least three times. B, Densitometric analyses of the iC3b 43 kDa band. Results are presented as the mean values ± SEM of three independent experiments in duplicate wells. The degradation of C3b was calculated by ratio of relative intensity of 43 kDa over 108 kDa. C, Uromodulin (UMOD)‐cFH enhanced the prevention of the Sheep erythrocytes from haemolysis. Adding 6 μg of cFH antibody induced nearly 100% haemolysis. The haemolysis was inhibited to 50% by adding 6 μg of cFH antibody and 3 μg exogenous cFH. Further, the haemolysis was inhibited up to 30% by introducing 5, 10 and 20 μg of UMOD. The control groups included UMOD (5, 10 and 20 μg) alone without cFH were also collected. The experiments were repeated at least three times

In this study, we further explored whether the existence of UMOD could accelerate C3b degradation mediated by cFH and factor I. The α‐chain (108 kDa) of C3b was cleaved into two fragments at 68 and 43 kDa. The ratio of 43 kDa over 108 kDa indicates the level of C3b degradation. The control group with UMOD alone (without cFH) was set up. With the extension of time, the group without UMOD presented no obvious increase of C3b degradation, but in the group with UMOD, an increase of C3b degradation was observed. The control group with UMOD alone present no degradation of C3b (Figure 1A, B).

The cFH protects sheep red blood cells (SRBCs) from haemolysis through inhibiting the activation of complement AP.^24^ In our test, we set up a system which led to nearly 100% haemolysis of the SRBCs by adding 6‐μg anti‐cFH antibody to the healthy serum. The haemolysis rate was reduced to 50% by adding exogenous 3 μg cFH in the system. When adding UMOD (5, 10 and 20 μg), the haemolysis rate was gradually reduced to 30%. We also set up control groups with various UMOD (5, 10 and 20 μg), but without cFH. The results showed that UMOD could not protect erythrocytes from haemolysis without cFH (Figure 1C).

### 3.2 Sialic acids on uromodulin mediated the binding of uromodulin and cFH

**FIGURE 2:**
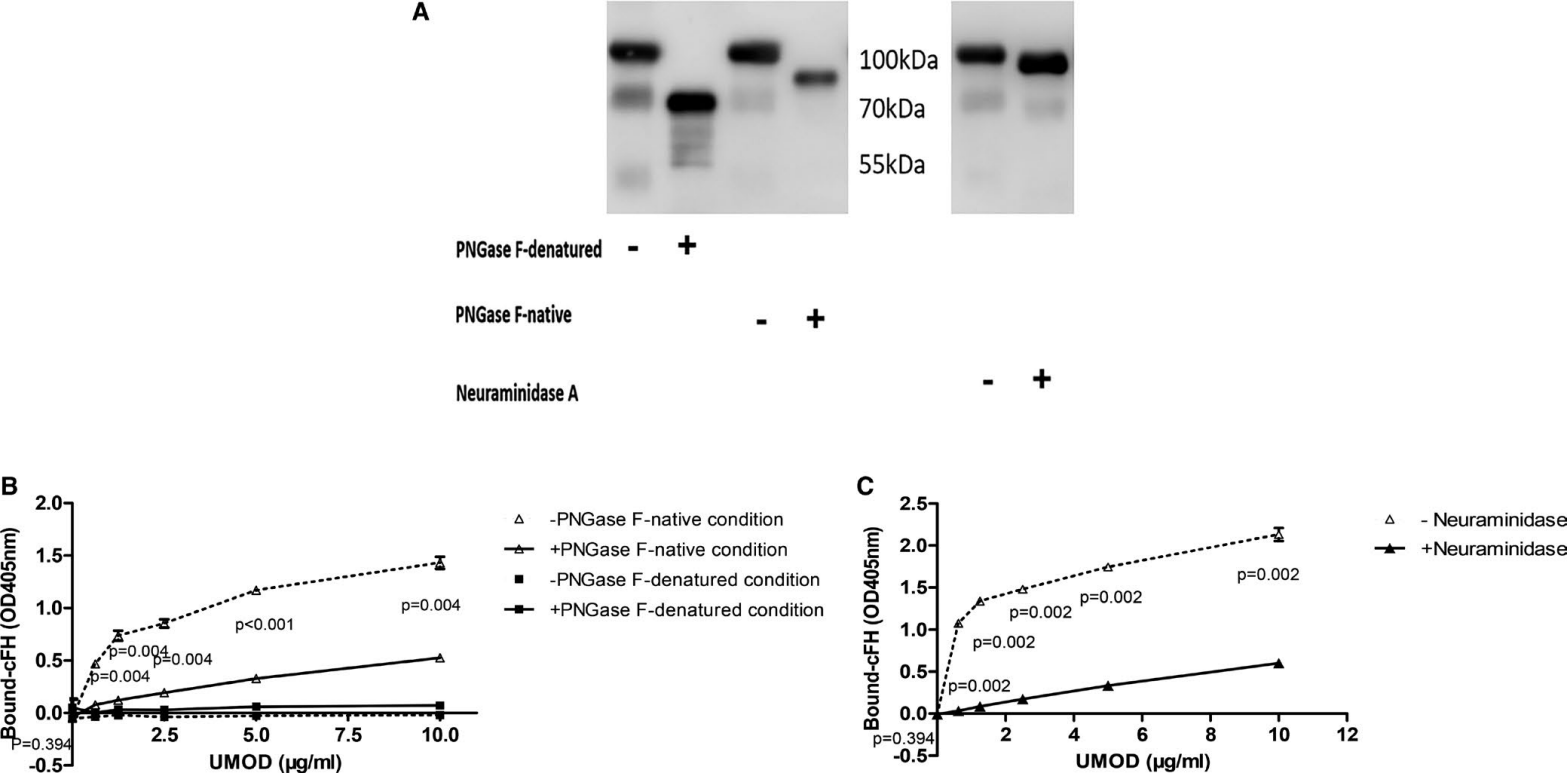
Sialic acids mediated the interaction of uromodulin and cFH. Uromodulin (UMOD) was treated with PNGase F or neuraminidase A to generate different kinds of de‐glycosylated forms. Then, the binding assay of de‐glycosylated UMOD and cFH was measured on 96‐well microtiter plates. The experiments were repeated at least three times. A, Uromodulin (UMOD) was treated with PNGase F (under denatured and native condition) and neuraminidase A, and then was analysed by western blotting with anti‐human UMOD antibody. B, UMOD pre‐treated with PNGase F (under native and denatured condition) bound immobilized cFH. C, UMOD pre‐treated with neuraminidase A bound immobilized cFH

UMOD was treated with PNGase F or neuraminidase A to generate different types of de‐glycosylated protein. Under denatured condition, most of the N‐glycans were removed by PNGase F, which led to about 30% decrease of the UMOD molecular weight (Figure 2A). Under denatured condition, UMOD lost the ability of binding cFH either with or without N‐glycans (Figure 2B). Under native condition, the N‐glycan chains were partially removed. When UMOD was treated with neuraminidase A under native condition, there was also a small band shifting, suggested partial removal of sialic acids (Figure 2A).

In order to exclude the influence of PNGase F and neuraminidase A on UMOD and cFH‐binding analysis. We used exclusion chromatography to remove the PNGase F (36 kDa) in reaction mixture and silver stain showed that most of the PNGase F was removed (Figure S2A). No difference was identified between filtered and not filtered de‐glycosylated UMOD‐cFH binding (Figure S2B). Filtering with exclusion chromatography lost most of UMOD protein due to the viscosity of UMOD and similar molecular weight of de‐glycosylated UMOD and neuraminidase. We tried another control experiment to demonstrate that no difference between the binding strength of UMOD with and without neuraminidase (not incubated at 37℃) to cFH (Figure S2C).

Under native condition, the removal of N‐glycan by PNGase F led to obviously decreased binding of UMOD to factor H (Figure 2B). We then found that removal of sialic acid components by neuraminidase A also obviously reduced the binding of UMOD to cFH (Figure 2C).

**FIGURE 3:**
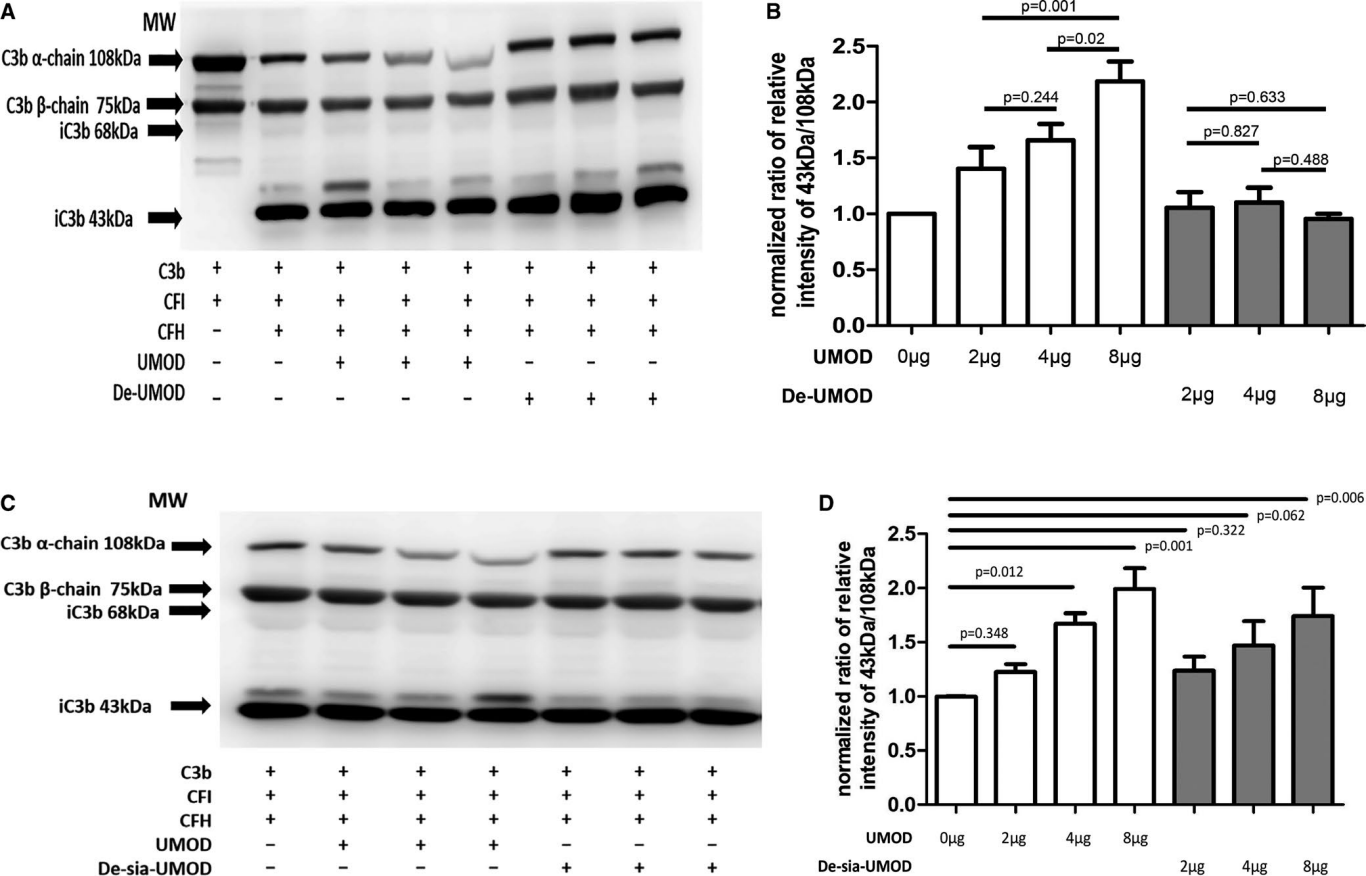
Uromodulin lost N‐glycans failed to enhance C3b degradation. The fluid reaction system, including C3b (3 μg), cFI (50 ng), cFH (0 or 0.5 μg), without or with uromodulin (UMOD) (2, 4 and 8 μg) and with de‐glycosylated or de‐sialylated UMOD (2, 4 and 8 μg) were incubated and samples were collected at 30 min (n = 3). The experiments were repeated at least three times. A, Western blotting of C3b and its lysis products with UMOD or de‐glycosylated UMOD. B, The degradation of C3b was calculated by normalized ratio of relative intensity of 43 kDa over 108 kDa. Normalization was carried out by ratio of the relative intensity of 43 kDa and 108 kDa of the sample dividing ratio of that of the control (with cFH and without UMOD). C, Western blotting of C3b and its lysis products with UMOD or de‐sialylated UMOD. D, The degradation of C3b was calculated by normalized ratio of relative intensity of 43 kDa over 108 kDa. Normalization was carried out by ratio of the relative intensity of 43 kDa and 108 kDa of the sample dividing ratio of that of the control (with cFH and without UMOD)

In cofactor activity assay of cFH, UMOD pretreated with PNGase F lost the ability to enhance C3b degradation compared with controls (Figure 3A, B).

When UMOD was pretreated by neuraminidase A, the ability of enhancing C3b degradation of UMOD decreased, but in large dose (8 μg UMOD) group, the de‐sia UMOD still enhanced the degradation compared with 0 μg UMOD group (Figure 3C, D).

### 3.3 Measurement of sia‐α (2,3) Gal/GalNAc in CKD patients

**FIGURE 4:**
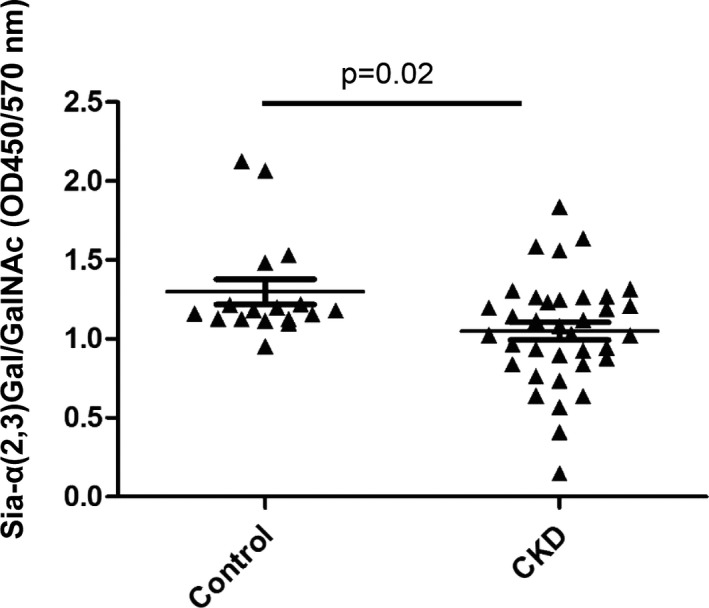
A decrease of Sia‐α (2,3) Gal/GalNAc was found in CKD patients. Sia‐α (2,3) Gal/GalNAc was specifically recognized by biotinylated Maackia ameurensis lectin II. The absorbance was read at OD450/570 nm and represented the concentration of α‐2,3 sialic acids on UMOD

We recruited 17 healthy controls and 36 CKD patients with average serum creatinine at 212.0 ± 102. 6 μmol/L. We collected their urine, purified the UMOD to exclude the influence of proteinuria. The same dosage of UMOD was used in the experiments. We measured α‐2,3 sialic acids level on UMOD by lectin‐ELISA in 17 healthy controls and 36 CKD patients. A decrease of α‐2,3 sialic acids on UMOD was observed (*P* = .02) in CKD patients (Figure 4 **)**.

### 3.4 The effect of pH on the interaction of UMOD and cFH

**FIGURE 5:**
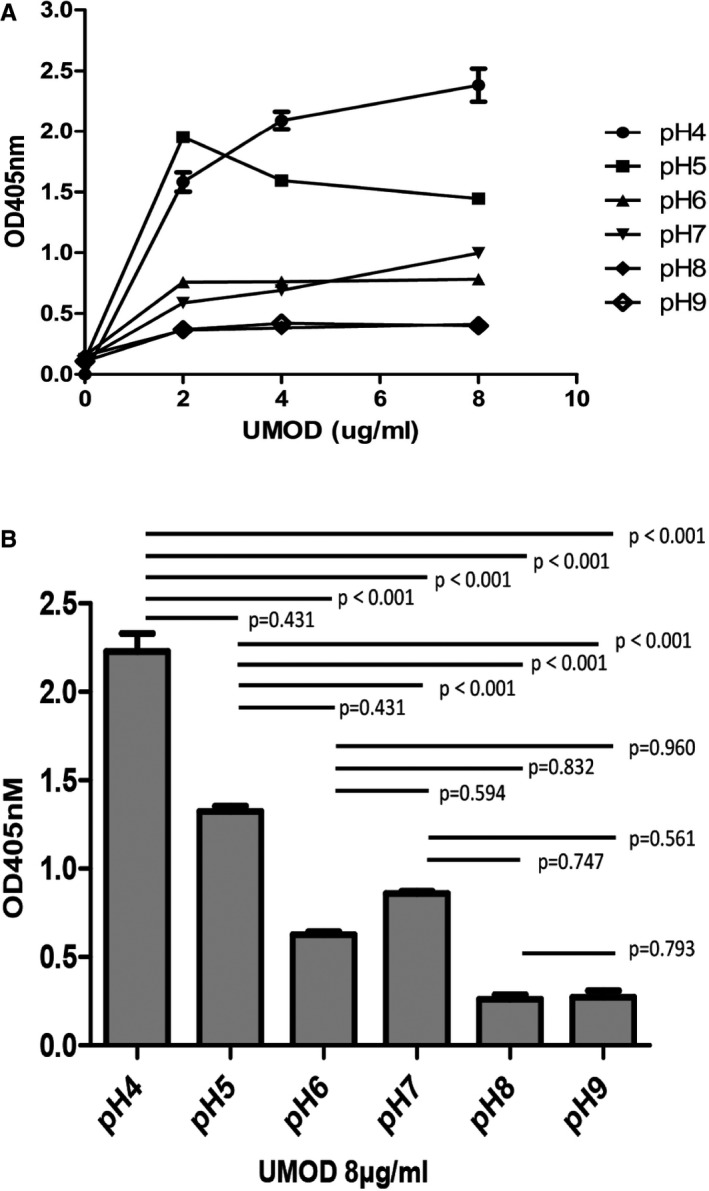
The binding between UMOD and cFH under different pH levels. A, The binding of different concentrations (0‐8 μg/mL) of UMOD with cFH at different pH were compared and presented. *P* (pH4:0‐2 μg/mL) < .001*; *P* (pH4:2‐4 μg/mL) < .001*; *P* (pH4:4‐8 μg/mL) = .027*; *P* (pH5:0‐2 μg/mL) < .001*; *P* (pH5:2‐4 μg/mL) < .001*; *P* (pH5:4‐8 μg/mL) = .005*; *P* (pH6:0‐2 μg/mL) < .001*; *P* (pH6:2‐4 μg/mL) = .847; *P* (pH6:4‐8 μg/mL) = .349; *P* (pH7:0‐2 μg/mL) < .001*; *P* (pH7:2‐4 μg/mL) = .001*; *P* (pH7:4‐8 μg/mL) < .001*; *P* (pH8:0‐2 μg/mL) < .001*; *P* (pH8:2‐4 μg/mL) = .302; *P* (pH8:4‐8 μg/mL) = .289; *P* (pH9:0‐2 μg/mL) = .002*; *P* (pH9:2‐4 μg/mL) = .434; *P* (pH9:4‐8 μg/ml) = .732. B, The concentration of UMOD was fixed at 8 μg/mL. The binding of UMOD with cFH in different pH value were compared and presented

Under different pH conditions (pH = 4‐9), the binding of UMOD and cFH was still dose‐dependent, increased with elevated UMOD dosage. With acidic condition (pH = 4), the dose‐dependent effect was clear and the binding was the strongest. While under alkaline environment (pH = 8‐9), the dose‐dependent effect was not obvious and the binding was the weakest (Figure 5A). The capacity of UMOD binding cFH was stronger under acidic conditions (pH = 4‐5) then under alkaline conditions (pH = 8‐9) at indicated UMOD dose (Figure 5B).

### 3.5 The effect of ion concentration on the interaction of UMOD and cFH

**FIGURE 6:**
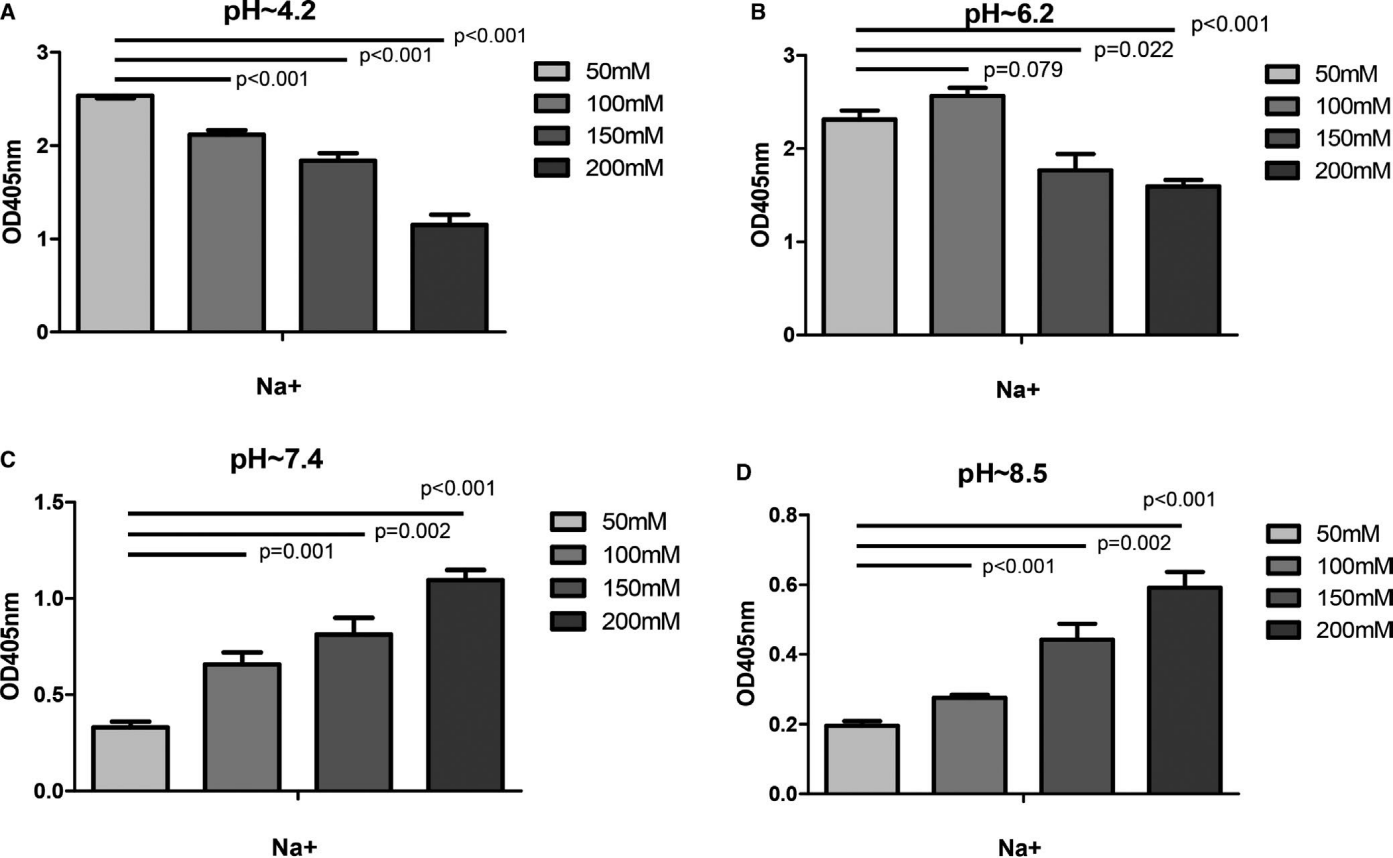
The binding between UMOD and cFH under different sodium and pH levels. The concentration of UMOD was fixed at 8 μg/mL. The binding of UMOD with cFH at different concentrations of sodium and pH were presented. A, When pH = 4.2. Sodium concentration is at 50, 100, 150, 200 mmol/L. B, When pH = 6.2. Sodium concentration is at 50, 100, 150, 200 mmol/L. C, When pH = 7.4. Sodium concentration is at 50, 100, 150, 200 mmo/L. D, When pH = 8.5. Sodium concentration is at 50, 100, 150, 200 mmo/L

Sodium concentration of 50, 100, 150 and 200 mmol/L was prepared at different pH. When the pH was fixed, the binding of UMOD and cFH changed with the concentration of sodium ion. The binding capacity decreased as sodium ion concentration increased at acidic condition. While under neutral and alkaline conditions, the binding got stronger as sodium concentration rose (Figure 6).

In order to explore the influence of calcium and magnesium, artificial urine was used as binding buffer and the pH was adjusted to 4.6, 5.4 and 6.0, respectively, by hydrochloric acid. The results showed that when the concentration of UMOD was constant, the binding ability of UMOD with cFH was increased as pH decreased. That was similar as the assay performed in PBS. Calcium and magnesium ions were removed from artificial urine, and pH of the urine was adjusted by hydrochloric acid. The binding capacity of UMOD and cFH was not influenced by calcium and magnesium (Figure S3).

## 4 DISCUSSION

We previously found that UMOD could bind with cFH and enhance the cofactor activity of cFH in the cleavage of C3b by cFI.^7^ And this work was also proved by Rhodes et al^25^ It is well known that cFH is the key regulator of complement AP, and the regulatory domain of cFH locates in N‐terminal short consensus repeat 1‐4 (SCR1‐4), whose function is to bind C3b, and to accelerate the degradation of C3 convertase.^7^, ^26^ C3b has α‐chain and β‐chain, and α‐chain (108 kDa) can be cleaved into 68 and 43 kDa. 68 kDa product is stable, while 43 kDa product is increasing with degradation of α‐chain.^27^ The ratio of 43 kDa over 108 kDa can indicate the degree of C3b degradation. The sheep erythrocytes haemolysis are typical tests to evaluate cFH function. C‐terminal of cFH binds negatively charged molecules, such as heparin and sialic acid, which makes cFH immobilized on the cell surface, and then the host cells can be protected from the injury of the over‐activation of complement AP, such as protecting erythrocytes from hemolysis.^28^

In our previous experiments,^7^ we confirmed binding between cFH and THP, with ELISA. We also did competition test and surface plasmon resonance (SPR) binding experiment to verify the combination. However, we only identified a dose‐dependent effect of UMOD on cFH at 30 minutes of co‐incubation. In this study, we measured the cleavage of C3b at 10, 20, 30 and 40 minutes after adding UMOD in co‐factor activity assays. The results showed that UMOD enhanced the degradation of C3b at each time point. And the cleavage of C3b kept increasing in the group with UMOD with time prolonged. The result indicated that UMOD could enhance the ability of cFH in degrading C3b, not only in a dose‐dependent way, but also a time‐dependent way. And we further demonstrated that this UMOD‐cFH binding did decrease the haemolysis rate of red blood cells.

With function tests of cFH, we further demonstrated that UMOD could enhance the cofactor function of cFH. The cFH is an important protective factor of cells through binding cell surface by c‐terminals.^28^ It can bind to renal tubular epithelial cells. When ischemia‐reperfusion injury occurs, cFH can inhibit the excessive activation of complement and reduce the damage of complement system to epithelial cells.^29^ cFH is related to the severity and prognosis of IgA nephropathy. The level of cFH in serum and urine of CKD patients is higher than that of normal people.^30^ UMOD is expressed on the cell surface of tubular cells, the interaction of UMOD and cFH strengthens the protective role of UMOD. The TAL and DCT segments face gradient changes of pH and ion concentration not only under physiological, but also pathological status.^8^, ^11^, ^12^ Our result indicated that the protective function of UMOD could adapt to the vigorous change of environment. Evidences have shown that progressive injuries of tubule‐interstitial tissue are closely related to activation of complement system.^31^ The study of Morita et al^32^ have suggested that the degree of intratubular complement activation correlates with impairment of renal function, and complement activation products excretion rate significantly decreases after sodium bicarbonate administration without affecting the level of plasma complement activation products. Decrease of pH value in renal tubules is involved in the activation of complements,^33^ and there are direct activation of complements, including AP when pH is between 5.5 and 8.^34^ Bicarbonate protects against complement‐mediated damage in the lumen by increasing the local pH^34^ and oral administration of sodium bicarbonate can attenuate the progress of CKD.^35^ In our study, the binding ability of UMOD with cFH was enhanced in acidic environment both in PBS and artificial urine, which indicated that UMOD‐cFH interaction was increased with the complement activation. In our study, the binding ability of UMOD with cFH was enhanced in acidic environment both in PBS and artificial urine. Evidences have shown that progressive injuries of tubule‐interstitial tissue are closely related to activation of complement system^31^ and decrease of pH value in renal tubules is involved in the activation of complements.^33^ When pH is between 5.5 and 8, there are direct activation of complements, including AP.^34^ Thus, oral prescription of sodium bicarbonate can attenuate the progress of CKD.^35^ Our findings suggested UMOD can play protective roles via enhancing activity of cFH in CKD.

Furthermore, we found that under acidic conditions, the binding of UMOD and cFH got stronger with the increased concentration of sodium ions. Conversely, in alkaline environment, it fell when pH value rose. A bunch of clinical studies have shown that dietary sodium restriction has protective effect on the kidney of patients with CKD.^36^, ^37^, ^38^ The mechanism of the effect may be associated with regulation of glomerular filtration and the reduction of local activation of renin‐angiotensin‐aldosterone system. Our study indicates that the decrease of urinary sodium excretion after restriction of dietary sodium might improve the binding ability of UMOD with cFH and inhibit complement activation. However, when pH changed, the effect of sodium ions on UMOD‐cFH binding changed conversely. It suggested that the restriction of sodium intake might be coordinated with alkalization of urine to maintain appropriate urinary pH value and tubular sodium concentration in clinical conditions.

On the other hand, UMOD is a highly N‐glycosylated protein, with sialic acids on the outer part.^16^ The carbohydrate structures of UMOD have different functions, such as binding microbial structure,^39^ lectin structure and extracellular matrix.^40^, ^41^ The high‐mannose side chain on UMOD mediates removal of type 1 fimbriated E. coli from urinary tract.^42^ In renal allograft recipients, disturbed glycosylation of UMOD was detected and the abnormal glycosylation of UMOD reduced its binding capacity to cytokines, and associated with tubular injury.^5^ Several evidences suggest that glycans of UMOD are essential for its immunosuppressive and cytokine‐binding activities.^43^, ^44^, ^45^ Under denatured condition, UMOD lost the ability of binding with cFH, so the native status was used to measure the influence of glycans. PNGase F can partly remove the glycans without selection. The loss of partial N‐glycans of UMOD under native condition, led to significant decrease of its binding with cFH. Previous studies also have found that the C‐terminus of cFH is responsible for its binding with sialic acid.^18^ The binding can be enhanced by the existence of surface‐deposited C3b.^19^, ^20^ Thus, sialic acid may be important for cFH to play the role in the context of immune surveillance. We further removed sialic acid on UMOD by neuraminidase A specifically. A decreased binding between UMOD and cFH was observed, and a lower ability of enhancing C3b degradation was also found. In this assay, the fact that no difference was identified between the binding strength of filtered and not filtered de‐glycosylated UMOD‐cFH suggested that the binding status was not influenced by PNGase F. Because of the viscosity of UMOD, filtering with exclusion chromatography lost a lot of UMOD protein. The molecular weight of de‐glycosylated UMOD is closer to that of neuraminidase; most of the UMOD was lost when separating them from mixed samples. We compared the binding of UMOD to cFH when they were mixed with or without neuraminidase at room temperature. No difference was found between the two groups. Furthermore, the binding assay was performed in PBS, and PBS was not the working buffer of neuraminidase A and PNGase F, which greatly reduced the effect of them.

We also identified that the level of α‐2,3 sialic acids of UMOD decreased in patients with CKD when compared with normal individuals. When CKD develops to four or five stage, patients usually suffer from tubular atrophy, renal fibrosis and decline of renal function, and the correlation between disease progression and primary disease is not as strong as early stage.^46^ Therefore, we did not specifically distinguish the primary diseases when we included the patients. This finding indicated that not only level of UMOD but also the status of sialic acid on UMOD may be involved in the progression of CKD. We only identified decreased α‐2,3 sialic acids, which is the type of sialic acids that can be recognized by cFH.^47^, ^48^

In summary, the binding of UMOD and cFH was regulated by local pH, local sodium concentration and sialic acid richness on UMOD. Although we detected sialic acid levels in CKD patients, most of our experiments were in vitro, more data from human is needed.

## CONFLICT OF INTEREST

The authors declare no conflicts of interest.

## AUTHOR CONTRIBUTION

**Lufeng Bai:** Data curation (equal); Investigation (equal); Writing‐original draft (equal). **Qiuyu Xie:** Data curation (equal); Investigation (equal); Writing‐original draft (equal). **Min Xia:** Investigation (equal). **Kunjing Gong:** Methodology (equal). **Na Wang:** Methodology (equal). **Yuqing Chen:** Conceptualization (lead); Funding acquisition (lead); Project administration (lead); Supervision (lead); Writing‐review & editing (equal). **Ming‐hui Zhao:** Writing‐review & editing (equal).

## Supporting information

Figure S1

Figure S2

Figure S3

Supplementary Material

## Data Availability

The data that support the findings of this study are available from the corresponding author upon reasonable request. The study was approved by the local ethics committee of Peking University First Hospital (protocol No. 2017[1280]). All participants in this study signed written informed consent forms.
