# Supplementary figures and images for: The importance of sialic acid, pH and ion concentration on the interaction of uromodulin and complement factor H
Source: J Cell Mol Med. 2021 Mar 31;25(9):4316–25. doi: 10.1111/jcmm.16492 (PMC8093974; doi:10.1111/jcmm.16492)

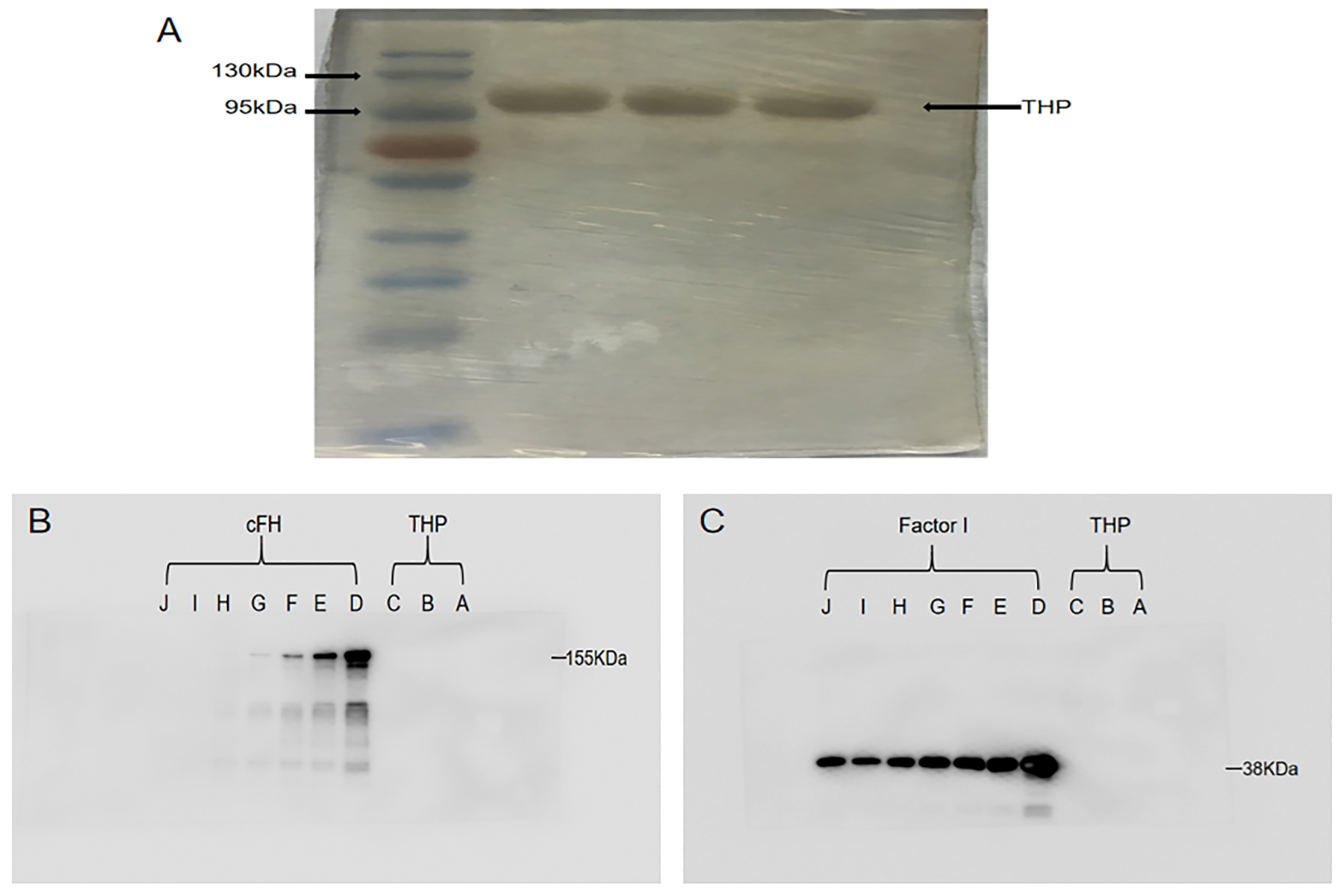

Supplement: Supplementary file 1 — Figure S1 [file JCMM-25-4316-s003.png]

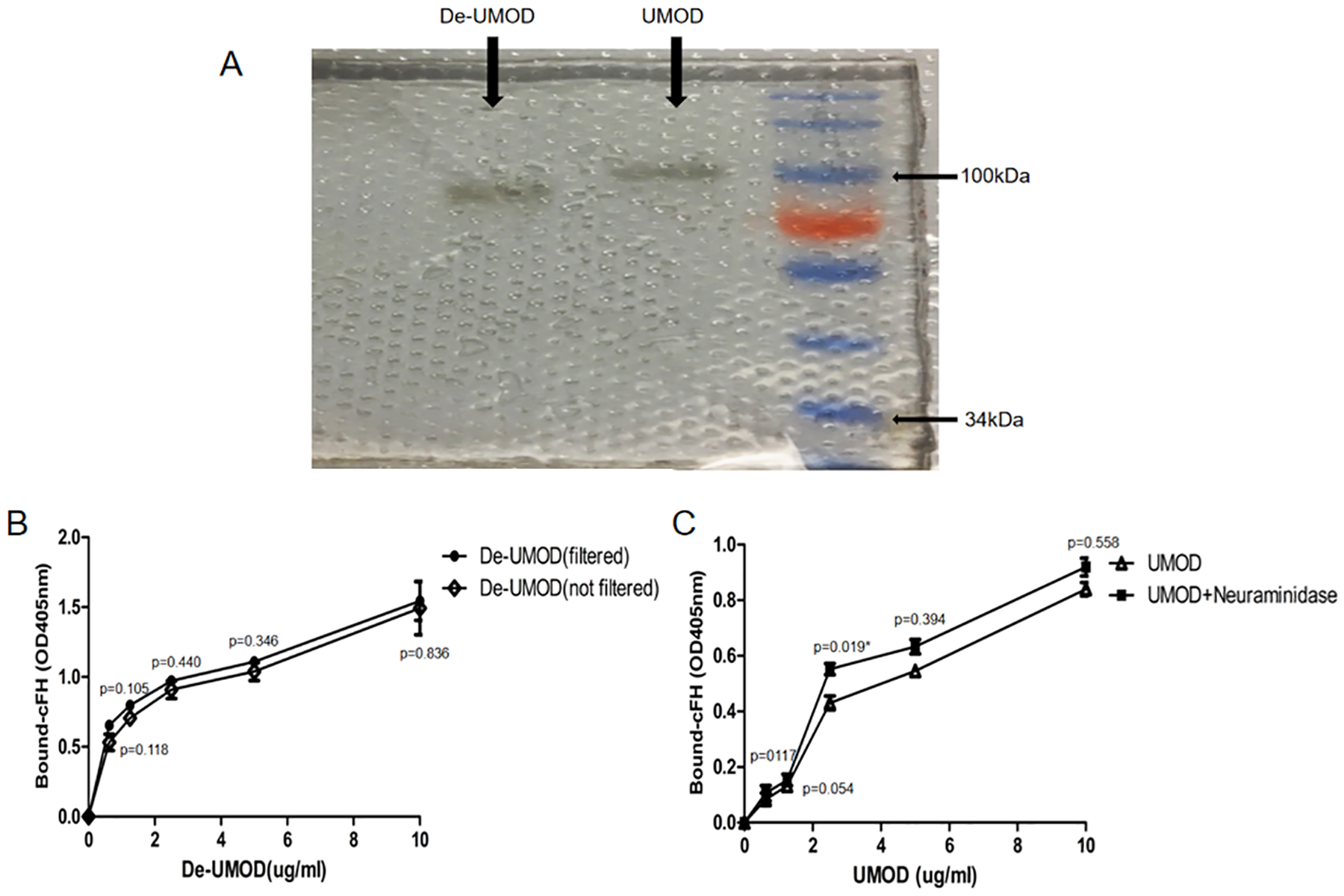

Supplement: Supplementary file 2 — Figure S2 [file JCMM-25-4316-s004.png]

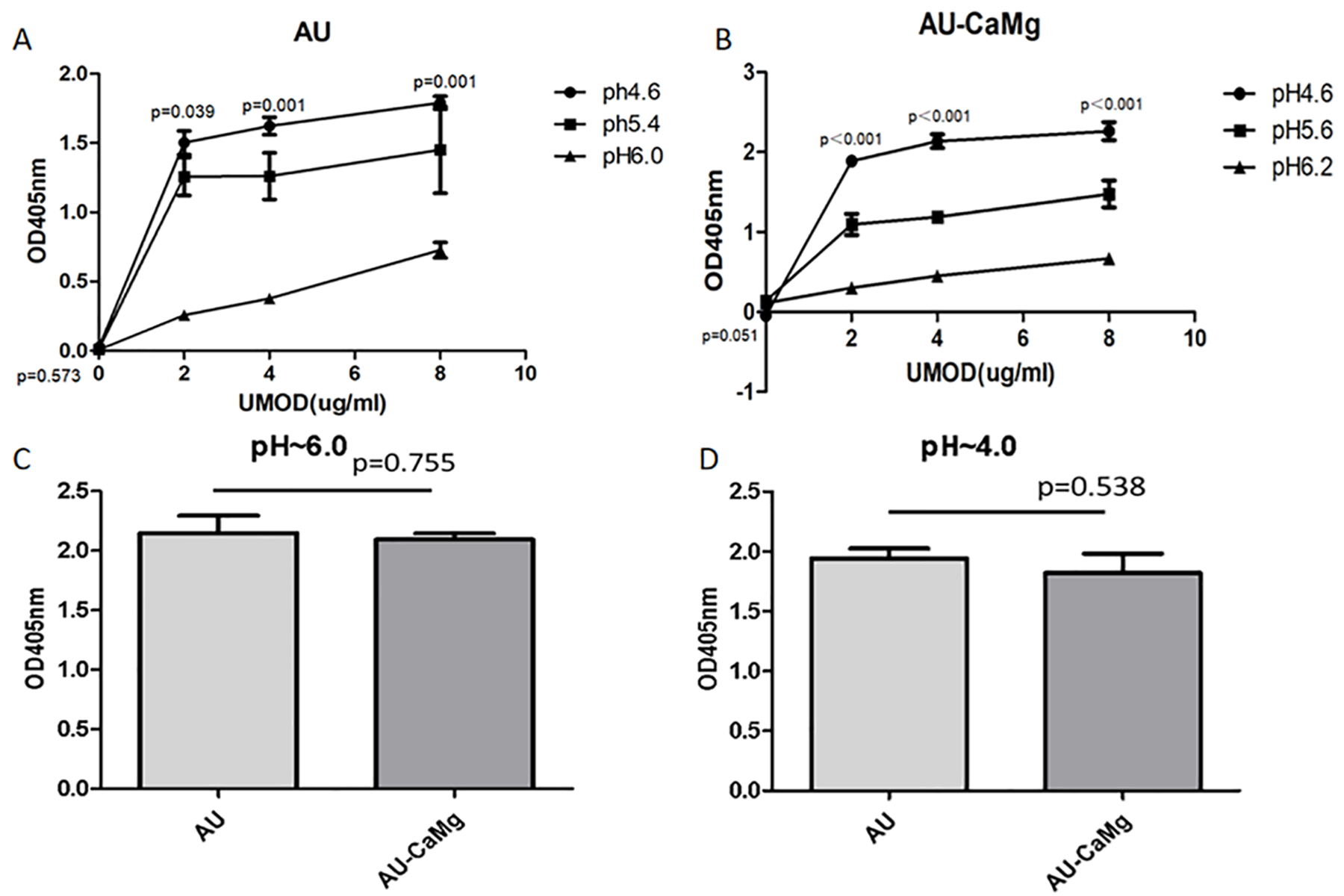

Supplement: Supplementary file 3 — Figure S3 [file JCMM-25-4316-s001.png]
