# Supplementary material for: The importance of sialic acid, pH and ion concentration on the interaction of uromodulin and complement factor H
Source: J Cell Mol Med. 2021 Mar 31;25(9):4316–25. doi: 10.1111/jcmm.16492 (PMC8093974; doi:10.1111/jcmm.16492)
Supplement: Supplementary file 4 — Supplementary Material [file JCMM-25-4316-s002.doc]

**Supplemental Figure 1. Identification of the purity of UMOD extracted from human urine.**

A: Silver staining of purified UMOD from human.

Samples in each lane contained 8 μg THP from 3 different healthy persons

B: Detection of complement factor H in purified UMOD from human.

Samples in lanes A to C contained 8 μg THP from 3 different healthy persons. Samples in Lanes D to J contained 1 μg, 0.5 μg, 0.25 μg, 0.125 μg, 0.0625 μg, 0.03125 μg, 0.015625 μg complement factor H respectively.

C: Detection of complement factor I in purified UMOD from human.

Samples in lanes A to C contained 8 μg THP from 3 different healthy persons. Samples in lanes D to J contained 1 μg, 0.5 μg, 0.25 μg, 0.125 μg, 0.0625 μg, 0.03125 μg, 0.015625 μg factor I respectively.

**Supplemental Figure 2.**

A: Silver staining of purified UMOD from human and filtered PNGase F-treated UMOD.

B: Uromodulin pre-treated with PNGase F (filtered and not filtered) bound immobilized cFH.

C: Uromodulin mixed with neuraminidase A (not incubated at 37℃) and purified uromodulin from human bound immobilized cFH.

**Supplemental Figure 3.**

The binding between UMOD and cFH in artificial urine.

A: AU: artificial urine, UMOD=0~8 μg/ml.

B: AU-CaMg: artificial urine without calcium and magnesium ions, UMOD=0~8 μg/ml.

C: When pH=4.0, UMOD=8 μg/ml.

D: When pH=6.0, UMOD=8 μg/ml.
